# Combination of diclofenac and aggressive hydration for the prevention of post-ERCP pancreatitis 

**Published:** 2018

**Authors:** Mehri Hajalikhani, Mohammad Hassan Emami, Mahsa Khodadoostan, Ahmad Shavakhi, Moeen Rezaei, Reza Soluki

**Affiliations:** 1 *Department of Gastroenterology and Hepatology, Faculty of Medicine, Isfahan University of Medical Sciences, Isfahan, Iran*; 2 *Gastrointestinal and Hepatobiliary Diseases Research Center, Poursina Hakim Research Institute for Health Care Development, Isfahan, Iran*; 3 *Medical Students’ Research Center, Faculty of Medicine, Isfahan University of Medical Sciences, Isfahan, Iran *

**Keywords:** Pancreatitis, Endoscopic retrograde cholangiopancreatography, Prevention, Inflammation, Diclofenac, Aggressive hydration

## Abstract

**Aim:**

To investigate whether aggressive hydration can increase the efficacy of prophylactic non-steroid anti-inflammatory drugs (NSAIDs) in prevention of post-ERCP pancreatitis.

**Background:**

NSAIDs are recommended for the prevention of PEP; however, whether aggressive hydration can have additional benefits in this regard is not known.

**Methods:**

Patients candidate for ERCP received either pre-procedural rectal diclofenac (100 mg) alone (n = 112) or in combination with aggressive hydration by lactate ringer’s (n = 107) as prophylactic method. PEP was defined based on increase in serum levels of pancreatic enzymes (from baseline to 24 hours following the procedure) accompanied with symptoms.

**Results:**

PEP was occurred in 3 patients in the diclofenac only group and in 1 patient in the diclofenac + hydration group with no significant difference (2.7% vs. 0.9%, P = 0.622). Serum amylase levels decreased over time in the diclofenac + hydration group but not in the diclofenac only group. Also, serum lipase levels decreased more rapidly over time in the diclofenac + hydration group compared to the diclofenac only group.

**Conclusion:**

Combination prophylactic therapy with NSAIDs plus aggressive hydration does not seem to have additional clinically important benefits in preventing PEP. Studies with larger sample of patients are required in this regard.

## Introduction

 Acute Pancreatitis following endoscopic retrograde cholangiopancreatography (ERCP) is the most common and serious adverse event associated with this procedure. The overall incidence of post-ERCP pancreatitis (PEP) is reported between 3 and 10% (1, 2). Incidence of PEP is higher in younger patients, females, and patients with suspected sphincter of Oddi dysfunction (SOD). Other patients-related risk factors associated with PEP include having normal serum bilirubin levels, prior PEP, and recurrent pancreatitis. Procedure-related factors increasing the risk of PEP include, but not limited to, biliary sphincter balloon dilation, difficult cannulation, pancreatic sphincterotomy, and pancreatic duct injection (3, 4). PEP results in considerable morbidity with the estimated costs of about 200 million dollars annually in the United States. The overall mortality rate associated with PEP is low and reported as 0.7% (2). Prevention of PEP is of great importance to reducing the morbidity and mortality associated with this procedure. 

Various strategies have been investigated for the prevention of PEP. Meta-analyses have shown that prophylactic pancreatic duct stenting reduces the incidence of PEP by about 60% (5, 6), and this method is recommended especially for patients at higher risk for PEP (7). Evidence also supports the effectiveness of nonsteroidal anti-inflammatory drugs (NSAIDs). Recent meta-analyses have shown that rectal administration of either indomethacin or diclofenac reduces the risk of PEP by about 40 to 45% (8, 9). However, studies do not support non-rectal administration of the NSAIDs for the prevention of PEP (10, 11). Therefore, routine administration of rectal NSAIDs has been recommended for all patients to reduce the incidence and severity of PEP (7, 12). Other preventive strategy is peri-procedural intravenous hydration. According to recent meta-analyses aggressive hydration with lactated ringers is an effective and safe method to reduce the risk of PEP with comparable efficiency as NSAIDs and pancreatic duct stenting (13, 14). 

Although current preventive strategies have significantly reduced the incidence of PEP, few studies have evaluated the possible benefits of combining different preventive strategies with various possible mechanisms of actions (15, 16). Such combined therapies may further reduce the incidence of PEP and costs associated with it. Therefore, we aimed to investigate the possible additional benefits of combining rectal NSAID and aggressive intravenous hydration with this hypothesis that such combination therapy is better than rectal NSAID alone in preventing PEP. 

## Methods

***Setting and Patients***

This controlled clinical trial was conducted on patient’s candidate for ERCP in the Zahraye Marzie charity Hospital during July 2017 to March 2018. The inclusion criteria were as follows: undergoing elective diagnostic/interventional ERCP, age between 18 and 70 years, and receiving pre-procedural rectal diclofenac with or without aggressive hydration for the prevention of PEP. Patients with any of the following conditions were not included into the study: contraindication for NSAIDs (e.g. recent gastrointestinal bleeding); chronic heart failure (NYHA class >2); hypoxemia (SaO2 <90%); renal failure (GFR <40 ml/min); liver failure/dysfunction (prolonged INR and low albumin level); evidence of fluid overload (e.g. pulmonary edema, hypo/hypernatremia); and pregnancy. The study was approved by the Ethical Committee of the Isfahan University of Medical Sciences (approval number: 196187) and informed consent was obtained from patients for using their data anonymously for research purposes. 

***Interventions***

ERCP was performed by a single endoscopist (MHE) for all patients based on standard cannulation techniques and using side-view endoscope (Pentax ED-3440T, Tokyo, Japan). At the time, the endoscopist had experience of performing 10000 ERCP during 18 years. 

All patients received the diclofenac sodium suppository (100 mg) about 30 minutes before the procedure. In addition, patients received either standard or aggressive intravenous hydration alternatively. Standard intravenous hydration was with lactate ringer’s 1.5 ml/kg/h during ERCP, continued for 8 hours following completion of the procedure. Aggressive hydration was with lactate ringer’s 3 ml/kg/h during ERCP plus a bolus dose of 20 ml/kg/h at the end of the procedure and then 3 ml/kg/h for 8 hours following completion of the procedure. 

***Measurements and Study outcomes***

Data were extracted from the hospital paper and electronic medical records. Demographic data included age, gender, and height and weight from which BMI (kg/m^2^) was calculated. Medical history included comorbidities (e.g. diabetes, hypertension), smoking, and previous pancreatitis. Procedural data were extracted from the ERCP reports. Laboratory data included complete blood count and liver function tests. Serum levels of amylase and lipase were measured at baseline (on admission), and then 2, 8, and 24 hours following completion of the procedure. Upper limits were considered as 100 u/l for serum amylase and 63 u/l for serum lipase according to the laboratory reference. 

The study primary outcome was the occurrence of PEP which was defined as increase in serum levels of pancreatic enzymes >3 times of the upper limit of normal accompanied with epigastric pain (or increase of pain in those who had pain before) persisting for at least 24 hours following the procedure. Mild pancreatitis was defined based on the absence of organ failure or local or systemic complications. Moderate pancreatitis was defined as having transient (resolved within 48 hours) organ failure and/or having local or systemic complications. Severe pancreatitis was defined as having a persistent organ failure not resolved within 48 hours (17). Other study outcomes were the post-procedural serum levels of amylase and lipase. 

***Statistical analysis***

Statistical analysis was performed using the SPSS software. The Kolmogorove-Smirnov test was used to check whether data are normally distributed (most data were not normally distributed). Independent sample t-test and Mann-Whitney U test were then applied for between group comparisons. Wilcoxon test was applied for within-group comparisons of changes in serum levels of pancreatic enzymes. A P value of <0.05 was considered significant in all analyses. 

## Results

Out of 341 evaluated patients, 219 patients were eligible to be included into the study including 112 patients who received NSAID alone and 107 patients who received combination therapy with NSAID + aggressive hydration. Characteristics of the patients are summarized in Table 1 and Table 2. The two groups were not similar regarding comorbidities and some laboratory tests at baseline. However, the number of PEP risk factors were the same between the two groups. 

**Table 1:** Comparison of demographic, medical, and laboratory data between the two groups

	NSAID n = 112	NSADI + Hydration n = 107	P value
Age, years	57.9 ± 9.7)	55.5 (10.9)	0.231
Female sex	55 (49.1%)	57 (53.3%)	0.589
BMI, kg/m^2^	24.6 ± 3.5)	24.7 (2.9)	0.809
Smoking	18 (16.1%)	26 (24.3%)	0.134
*Comorbidities*			
Hypertension	44 (39.3%)	27 (25.2%)	0.031
Coronary artery disease	12 (10.8%)	20 (18.7%)	0.126
Diabetes	39 (34.8%)	24 (22.4%)	0.052
Total comorbidities	1 [0 to 1]	0 [0 to 1]	0.049
*Laboratory data*			
WBC, 10^3^/mL	8.0 ± 2.9	8.0 ± 3.1	0.987
RBC, 10^3^/mL	4.7 ± 0.8	4.8 ± 0.6	0.159
Hemoglobin, gr/dL	12.9 ± 1.7	14.2 ± 1.9	<0.001
Platelets, 10^3^/mL	246.7 ± 108.9	245.2 ± 76.8	0.620
AST, U/L	51.5 ± 44.3	79.7 ± 81.5	0.223
ALT, U/L	64.1 ± 66.8	110.0 ± 107.9	0.004
ALKP, U/L	454.0 ± 341.1	653.8 ± 560.0	0.007
Total bilirubin, mg/dL	3.1 ± 4.9	5.4 ± 6.5	0.001
Direct bilirubin, mg/dL	1.6 ± 3.2	1.2 ± 1.4	0.823

Data are presented as mean ± SD, number (%), and Median [IQ25% to IQ75%]

**Table 2:** Comparison of procedural data and findings between the two groups

	NSAID n = 112	NSADI + Hydration n = 107	P value
Duration, minute	30.4 ± 10.7	30.3 ± 10.0	0.758
Biliary sphincterotomy	3 (2.7%)	1 (0.9%)	0.622
Precut sphincterotomy	24 (21.4%)	23 (21.5%)	>0.999
Balloon Dilation	38 (33.9%)	45 (42.1%)	0.265
Metallic biliary stent	24 (21.4%)	15 (14%)	0.162
Plastic biliary Stent	4 (3.6%)	5 (4.7%)	0.744
Pancreatic duct cannulation	9 (8%)	9 (8.4%)	>0.999
Pancreatic sphincterotomy	8 (7.1%)	7 (6.5%)	>0.999
Wide sphincterotomy	37 (33%)	28 (26.2%)	0.302
Pancreatic stent	3 (2.7%)	3 (2.8%)	>0.999
*ERCP findings*			
CBD stone	48 (42.9%)	51 (47.7%)	0.499
CBD stricture	32 (28.6%)	24 (22.4%)	0.353
SOD	22 (19.6%)	16 (15%)	0.378
Cholangiocarcinoma	22 (19.6%)	24 (22.4%)	0.623
Number of PEP risk factors a	1 [1 to 2]	2 [1 to 2]	0.679

Data are presented as mean ± SD, number (%), and Median [IQ25% to IQ75%].

a female sex, age < 40 years, sphincter of Oddi dysfunction, normal bilirubin, pancreatogram, pancreatic sphincterotomy, papillary balloon dilation, precut sphincterotomy, ampullectomy

**Table 3:** Comparison of study outcomes between the two groups

	NSAID n = 112	NSADI + Hydration n = 107	P value
PEP	3 (2.7%)	1 (0.9%)	0.622
Amylase > 3 times normal	4 (3.6%)	1 (0.9%)	0.369
Lipase > 3 times normal	3 (2.7%)	1 (0.9%)	0.622

Data are presented as number (%)

Study outcomes are summarized in the Table 3. Mild PEP was occurred in 3 patients (2.7%) of the NSAID group and 1 patient (0.9%) of the NSAID + hydration group, but this difference was no statistically significant. There was no significant difference between the two groups in the number of patients with increase in serum levels of pancreatic enzymes >3 times the upper limit of normal. 

**Figure 1:**
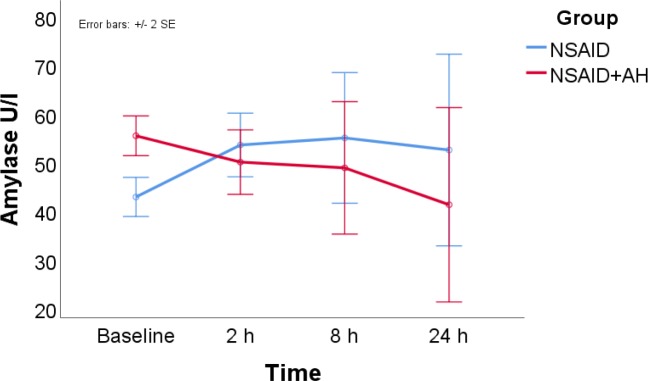
Serum amylase level from before to 24 hours after the procedure

**Figure 2:**
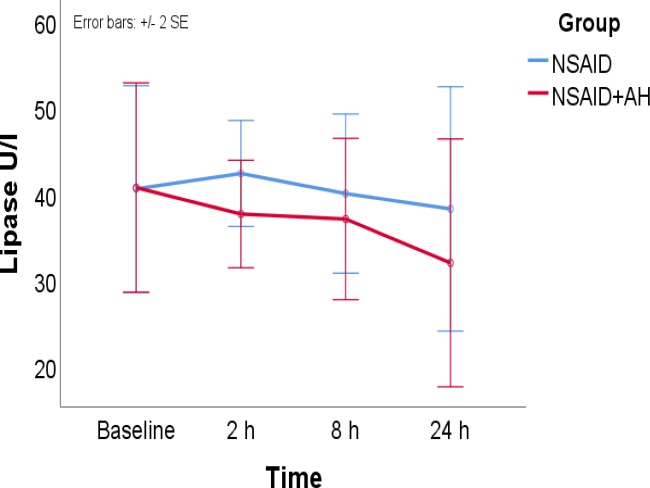
Serum lipase level from before to 24 hours after the procedure

The only significant difference between the study groups was the trend of changes in serum levels of pancreatic enzymes from baseline to 24 hours after ERCP (Figure 1 and Figure 2). In patients who received NSAID + hydration, serum levels of amylase was lower at 2 hours, 8 hours, and 24 hours after ERCP compared with baseline levels (P <0.001 at all levels). In contrast, in patients who only received NSAID, serum levels of amylase was higher at 2 hours (P = 0.005), 8 hours (P = 0.027), and 24 hours (P <0.001) after ERCP compared with baseline levels (Figure 1). Also, in patients who received NSAID + hydration, serum levels of lipase was lower at 2 hours, 8 hours, and 24 hours after ERCP compared with baseline levels (P <0.001 at all levels). 

In contrast, in patients who only received NSAID, serum levels of lipase was lower only at 24 hours after ERCP (P <0.001) compared with baseline levels (Figure 1).

## Discussion

Results from several studies support the efficacy of rectal NSAIDs in the prevention of PEP (7, 8). 

However, whether rectal NSAIDs should be used for all patients regardless of the risk of PEP is yet controversial. The study by Levenick *et al*. on 449 unselected patients found no reduction in PEP with rectal indomethacin compared to placebo (7.2% vs. 4.9%) (18). In contrast, a recent large randomized trial with 2600 patients showed that routine pre-procedural rectal indomethacin is better than selective (risk-stratified) post-procedural intervention in preventing PEP (4% vs. 8%) without increasing risk of bleeding (19). The efficacy of rectal diclofenac and indomethacin also seems comparable. In the study by Mohammad Alizadeh *et al.,* incidence of PEP was 4% with diclofenac (100 mg) and 5.8% with indomethacin (100 mg) compared to 15.9% with naproxen (20). In our study, the overall incidence of PEP was low (1.8%) which supports the benefit of routine administration of rectal NSAIDs for the prevention of PEP.

Hydration with large volume (aggressive hydration) is recommended in the early management of acute pancreatitis regardless of the etiology (21). In the context of PEP, several studies have shown the effect of aggressive hydration for the prevention of PEP. In the study by Park and colleagues on 395 patients, the incidence of PEP was lower with aggressive hydration (using lactate ringer’s) compared to standard hydration (1.6% vs. 11.6%) (22). Similar results are reported by Buxbaum *et al*. in a smaller study (23). Although NSAIDs and aggressive hydration are separately recommended for the prevention of PEP, little is known about the possible benefits of combination prophylactic therapies. In the study by Mok and colleagues, patients at high risk for PEP were randomized to four groups of normal saline + placebo, normal saline + indomethacin, lactate ringer’s + placebo, or lactate ringer’s + indomethacin. Patients in the latter group (combination therapy) had the lowest incidence of PEP (6%) compared to the other groups (13-21%) (16). In contrast to this study, in our study the incidence of PEP was lower in patients received the combination therapy with diclofenac and hydration compared to the diclofenac alone, although this difference was not statistically significant. Of note, in the study of Mok and colleagues, patients received 1 liter of lactate ringer’s before ERCP while in our study patients received the fluid based on weight during ERCP and as a bolus at the end of the procedure. Differences in volume and timing of fluid administration may explain part of the differences in the studies’ results (24). Further studies are yet required in this regard before a clear conclusion can be made. Considering the burden and possible risk of volume overload with aggressive hydration (13) we cannot yet recommend combination therapy with hydration and rectal NSAIDs for the prevention of PEP. Aggressive hydration can however be considered as an alternative prophylactic treatment for those with contraindications against NSAIDs.

The strength of this research study was that all ERCPs were performed by a single skilled gastroenterologist. However, there are also some limitations to this study. Since both groups had received a highly effective intervention the incidence of PEP was low and therefore a much larger sample size was required to compare the study primary outcome between the two groups. Combination prophylactic therapy with rectal diclofenac plus aggressive hydration with lactate ringer’s does not seem to have additional clinically important benefits in preventing PEP. Studies with larger sample of patients are required in this regard.
